# Safeguarding microbial biodiversity: microbial conservation specialist group within the species survival commission of the International Union for Conservation of Nature

**DOI:** 10.1093/ismejo/wraf239

**Published:** 2025-11-20

**Authors:** Jack A Gilbert, Amber Hartman Scholz, Maria Gloria Dominguez Bello, Lise Korsten, Gabriele Berg, Brajesh K Singh, Antje Boetius, Fengping Wang, Chris Greening, Kelly Wrighton, Seth R Bordenstein, Janet Jansson, Jay T Lennon, Valeria Souza, Sarah M Allard, Torsten Thomas, Don Cowan, Thomas W Crowther, Nguyen Nguyen, Lucy Harper, Louis-Patrick Haraoui, Suzanne L Ishaq, Margaret McFall-Ngai, Kent H Redford, Raquel Peixoto

**Affiliations:** Scripps Institution of Oceanography, University of California San Diego, La Jolla 92093, United States; Applied Microbiology International, Cambridge, CB1 2LA, United Kingdom; Leibniz Institute DSMZ German Collection of Microorganisms and Cell Cultures, Inhoffenstraße 7B, Braunschweig 38124, Germany; Department of Biochemistry and Microbiology, Rutgers University, New Brunswick, NJ 08901, United States; Department of Anthropology, Rutgers University, New Brunswick, NJ 08901, United States; Department of Plant and Soil Sciences, University of Pretoria, Hatfield, Pretoria 0028, South Africa; Department of Science and Innovation-National Research Foundation Centre of Excellence Food Security, Pretoria 0001, South Africa; Graz University of Technology, Environmental Biotechnology, Graz 8010, Austria; Leibniz Institute for Agricultural Engineering and Bioeconomy Potsdam and University of Potsdam, 14469, Germany; Hawkesbury Institute for the Environment, Western Sydney University, Penrith 2751 NSW, Australia; Monterey Bay Aquarium Research Institute, Moss Landing, CA 95039, United States; International Center for Deep Life Investigation, State Key Laboratory of Submarine Geoscience, School of Oceanography, Shanghai Jiao Tong University, 200240, China; Department of Microbiology, Biomedicine Discovery Institute, Monash University, VIC 3800, Australia; Securing Antarctica’s Environmental Future, Monash University, VIC 3800, Australia; Department of Soil and Crop Sciences, Colorado State University, Fort Collins, CO 80523, United States; One Health Microbiome Center and Departments of Biology and Entomology, The Pennsylvania State University, University Park, PA 16802, United States; Applied Microbiology International, Cambridge, CB1 2LA, United Kingdom; Pacific Northwest National Laboratory, Richland, WA 99354, United States; Indiana University, Bloomington, IN 47405, United States; American Society for Microbiology, American Academy of Microbiology, Washing DC 20036-2904, United States; Departamento de Ecología Evolutiva, Instituto de Ecología, Universidad Nacional Autónoma de México, Ciudad de México 04510, México; Centro de Estudios del Cuaternario de Fuego-Patagonia y Antártica (CEQUA), Punta Arenas 6200000, Chile; Scripps Institution of Oceanography, University of California San Diego, La Jolla 92093, United States; Centre for Marine Science and Innovation & School of Biological, Earth and Environmental Sciences, The University of New South Wales, Sydney, NSW 2052, Australia; Centre for Microbial Ecology and Genomics, Department of Biochemistry, Genetics and Microbiology, University of Pretoria, Pretoria, 0002, South Africa; King Abdullah University of Science and Technology, Thuwal, 23955, Saudi Arabia; American Society for Microbiology, American Academy of Microbiology, Washing DC 20036-2904, United States; Applied Microbiology International, Cambridge, CB1 2LA, United Kingdom; Department of Microbiology and Infectious Diseases, Faculty of Medicine and Health Sciences, Université de Sherbrooke, Québec, J1N 3C6, Canada; Centre de recherche Charles-Le Moyne, CISSS Montérégie-Centre, Greenfield Park, QC, J4V 2G9, Canada; Humans & The Microbiome Program, Canadian Institute for Advanced Research, Toronto, ON, M5G 1M1, Canada; School of Food and Agriculture, University of Maine, Orono, ME 04469, United States; Microbes and Social Equity Working Group, Orono, ME 04469, United States; NOVA Institute for Health, Baltimore, MD 21231,United States; Division of Biology and Biological Engineering, Carnegie Science, California Institute of Technology, Pasadena, CA 91125, United States; Archipelago Consulting, Portland, ME 04101, United States; King Abdullah University of Science and Technology, Thuwal, 23955, Saudi Arabia; International Society for Microbial Ecology (ISME), Arnhem 6708, The Netherlands; International Coral Reef Society (ICRS), Tavernier, FL 33070, United States

## Introduction

**Figure 1:**
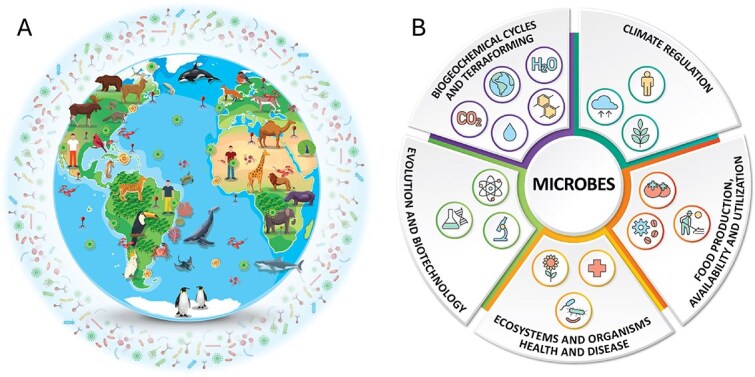
***We live in a microbial world***. (**A**) *Microbial distribution*: Microbes are ubiquitous, inhabiting every known ecosystem; from deep oceans, polar ice, and arid deserts to soils, freshwater, and the atmosphere, and forming intimate associations with all forms of life, including plants, animals, and humans - as well as among themselves. The figure illustrates the global distribution of microbial life across diverse environments and as symbionts. (**B**) *The central role of microbes in planetary systems and human society*. The ubiquitous distribution showed in Fig. 1A underscores the central importance of microbes in sustaining life on Earth and enabling future planetary habitability. They drive biogeochemical cycles and could be harnessed for terraforming; regulate climate through carbon sequestration, greenhouse gas production and consumption, and nutrient cycling; influence evolution and are engines of genetic innovation; and underpin biotechnology across health, industry, and environmental sectors. Microbes are essential for maintaining ecosystem and organism health, yet can also cause disease. They are critical to food production, availability, and utilization, from agriculture and aquaculture to fermentation and nutrient recycling. This interconnected network of microbial functions highlights their unparalleled importance in both natural processes and applied solutions for global challenges. Some of the vectors in Fig. 1A were designed by macrovector/Freepik.

Microorganisms—including microscopic single-cell and multicellular life—form the biological foundation of life on Earth. They regulate biogeochemical cycles, control climate-relevant gas fluxes, and underpin the health of all multicellular organisms [1–4] (Fig. 1A and B). Microbial communities drive key functions such as carbon sequestration, nitrogen fixation, gas cycling, soil fertility, marine productivity, and host digestion and immunity, making them indispensable to ecosystems, economies, and public health [5] (Fig. 1B). Despite their crucial roles, microbes and their impacts on visible life remain critically underrepresented in conservation science and policy [6]. Global conservation frameworks, including the International Union for Conservation of Nature (IUCN) Red List of Threatened Species and the Convention on Biological Diversity (CBD), have focused overwhelmingly on visible macroscopic taxa, neglecting microbial diversity (except the recent inclusion of a fungal SSC) despite mounting evidence of its vulnerability [7–13] and importance for global conservation action [5, 14, 15]. Similarly, major health frameworks such as One Health [16] have largely overlooked environmental microbial communities, especially those in soils and aquatic systems, despite their foundational importance for ecosystem stability and human well-being [17, 18]. Furthermore, this knowledge gap poses a significant risk as the loss of microbial diversity may destabilize ecosystem functions and compromise the success of broader conservation strategies [6, 11].

**Figure 2:**
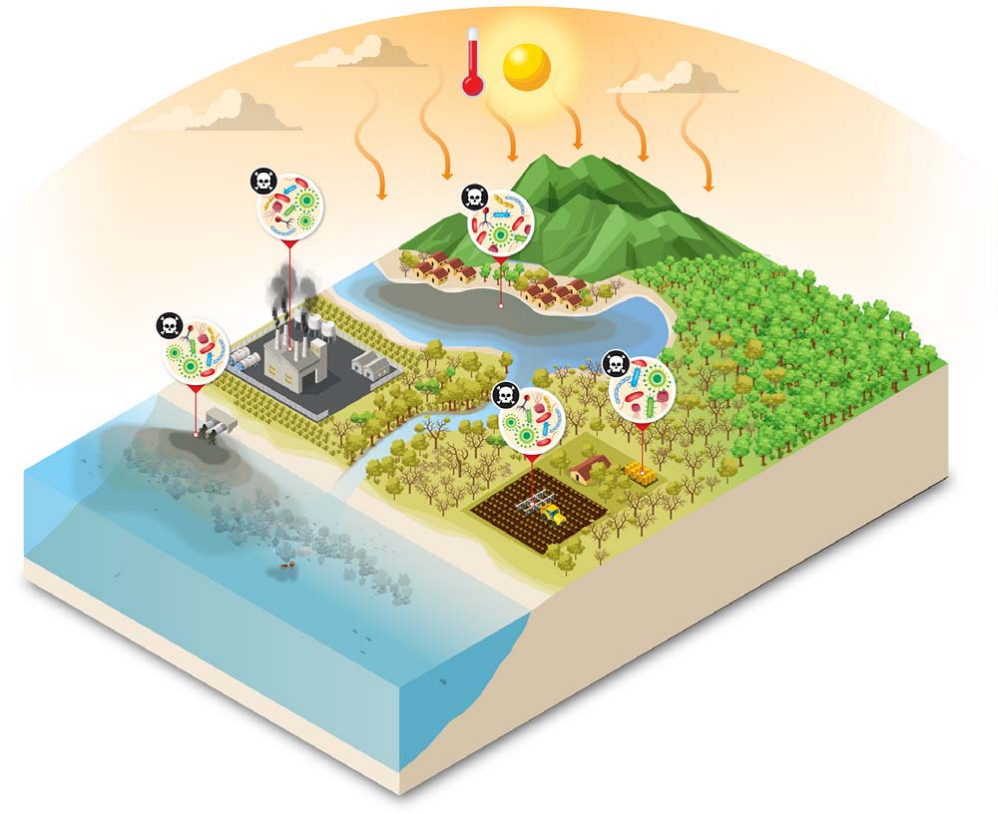
***There is no conservation without microbial conservation***: Anthropogenic stressors and loss of microbial diversity drive ecosystem degradation and pathogen proliferation across connected biomes. In absence of diversity and beneficial microbiota, terrestrial, coastal, and marine habitats are increasingly linked by the spread of harmful microorganisms fueled by pollution, habitat destruction, and climate change. Deforestation, agricultural runoff, chemical contamination, and industrial waste disrupt microbial community balance, leading to the replacement of beneficial microbes by pathogenic taxa. These pathogens circulate among ecosystems and hosts, exacerbating biodiversity loss, impairing organism health, and threatening ecosystem services and, ultimately, human health. Some of the figure vectors were designed by macrovector/Freepik.

## The case for microbial conservation

Microbial ecosystems and individual taxa are increasingly imperiled by a range of anthropogenic pressures that disrupt their structure, function, and, in the case of host-associated taxa, intergenerational transmission. These losses threaten ecosystem stability, human health, food security, and the climate resilience of individual ecosystems and the planet. Given microbes’ foundational roles in all life-supporting processes, the decline and/or disruption of microbiological communities may have as unpredictable and perilous consequences as macrobiological extinctions. Loss of certain microbial taxa could scale to community and food web disruptions and thereby potentially impact on climate regulation, collapsing nutrient cycling and soil fertility, impairing essential ecosystem services, increasing the risk of disease outbreaks, and ultimately diminishing global biodiversity and evolutionary potential. Among the primary drivers of microbial richness decline are habitat destruction, climate change, pollution, and human-mediated homogenization of natural environments. We will briefly outline these threats here.

Processes causing large-scale habitat destruction include de- forestation, agricultural intensification, glacial and permafrost melt, sea-ice melt, coral reef degradation, bottom trawling, and potentially also deep-sea mining (Fig. 2). These processes all alter microbial communities and potentially eliminate niche-specialist taxa. These disruptions impair ecological functions such as nutrient cycling, carbon sequestration, and host–microbe interactions that determine holobiont form, function, and variation. For example, the disruption of human ancestral habitat toward urbanized environments can lead to disruptions in holobiont compositions and increases in inflammation and chronic stress of hosts [19]. Also, deep-sea mining endangers microbial assemblages responsible for metal cycling and primary productivity in hydrothermal vents [20, 21].

Climate change further destabilizes microbial communities by altering temperature, moisture, and pH regimes across diverse systems [22]. Melting glaciers, permafrost, and sea ice threaten cold-adapted microbes evolved over millennia. In soils, warming reduces microbial carbon storage and increases greenhouse gas emissions [5, 23]. Marine warming and acidification, in turn, disrupt coral microbiomes, accelerating reef decline [24–26].

Pollution from antibiotics, pesticides, heavy metals, and plastics disturbs microbial networks in environmental and holobiont contexts [27–29]. Such exposure potentially eliminates beneficial microbes while selecting for resistant and pathogenic strains, thereby undermining ecosystem services and host immunity.

Human-mediated homogenization via urbanization, industrial food systems, and erosion of indigenous lifeways is also collapsing microbial diversity [27, 30, 31]. The shift from traditional microbiome-rich environments to sanitized lifestyles hampers intergenerational transmission and biocultural heritage [32, 33]. Studies have documented dramatic losses of keystone microbial taxa in industrialized populations, with lasting con- sequences for immunity, metabolism, and neurological health [27, 34, 35].

While it is true that microbes have demonstrated extraordinary genetic resilience through Earth’s mass extinction events, this perspective overlooks several critical points. First, the current loss of microbial diversity is occurring at an unprecedented rate and is largely anthropogenic—driven by industrialization, land-use change, climate warming, antibiotic overuse, and pollution. Unlike past extinctions caused by natural cataclysms in the current era of the Anthropocene, human activity is the principal force behind microbial decline. Second, while microbial communities are naturally dynamic, can adapt and reorganize, the rapid erosion of diversity weakens ecosystem resilience, making systems more vulnerable to perturbations and less able to recover from stress. Functional redundancy exists, but only up to a point—continued loss reduces the pool of traits available to support ecosystem functions, especially under changing environmental conditions. Finally, the notion that technological substitutes can replace microbial ecosystem services underestimates the complexity and interdependence of microbial networks in biogeochemical cycles, human and animal health, and climate regulation. These services are not easily replicable, and assuming we can engineer our way out of biodiversity loss risks complacency in the face of potentially irreversible ecological consequences.

It would seem axiomatic that preserving microbial ecosystems is not merely academic but is instead a prerequisite for achieving global conservation and restoration aims such as to protect 30% of Earth’s ecosystems by 2030. This is especially true for interactions between microbes and macrobes (e.g. animals and plants). Therefore, while we must push for microbial conservation, we can also improve global conservation efforts through integrating microbial knowledge into existing conservation strategies. For example, microbial restoration holds promise for ecosystem resilience: probiotics are deployed to reduce bleaching of coral reefs [10], soil microbiomes are central to regenerative agriculture [23], and microbiota interventions are tested for the recovery of amphibians, bats, and pollinators impacted by microbial dysbiosis [36], and also in improving human health. Including microbiology as a category of living systems can also significantly improve the success in strategies proposed to address the UN sustainability goals [5].

## The IUCN microbial conservation specialist group (MCSG)

In response to the growing recognition that microbial species and ecosystems are both foundational to life and increasingly imperiled, the IUCN Species Survival Commission (SSC) has formally established the MCSG, the first body within the IUCN dedicated to the stewardship of all microbial life. An existing Fungal SSC has already led the charge by developing an IUCN red list with *>*1000 fungal taxa. This group represents a critical expansion of the IUCN’s mandate and technical competency, acknowledging that conservation cannot succeed without assessing and protecting the microbial communities that sustain biodiversity, ecosystem function, and human health. The MCSG has received funding from private foundations, Applied Microbiology International, and the International Society for Microbial Ecology to support the development of the following strategy over the next 18 months. Here follows the mission statement, strategic objectives, integration with other IUCN programs, and a call for engagement.

*Mission Statement:* To safeguard and foster microbial species and their function across Earth’s ecosystems, recognizing microbes as the foundation of life and a cornerstone of planetary, macrobial species, and human health.

*Strategic Objectives:* The MCSG aims to coordinate with all other relevant IUCN programs to fully integrate microbial perspectives into the IUCN Species Conservation Cycle through five core functions: Assessment, Planning, Action, Networking, and Communication & Policy:

1. Assessment. Microbial life has historically been excluded from conservation assessment frameworks due to taxonomic biases, challenges in taxonomy and in defining what constitutes a microbial species, its invisibility in ecosystems, the lack and complexity of baseline data for microbial communities, as well as of ecological concepts to measure risk of loss. The MCSG will address this gap by pioneering tools and standards to evaluate microbial conservation status:

- Map microbial conservation hotspots and their threats, including unique and vulnerable microbial ecosystems such as Antarctic cryptoendoliths, hypersaline mats, cryosphere, and animal and plant- associated microbiomes; and define and identify endangered microbial populations (individual species) and communities (assemblages of different species), especially those most consequential for sustaining critical ecosystem services.
- Map the worlds biobanks and culture collections and assess methods and technologies to advance baseline studies and time series of microbial communities, especially in under sampled areas, such as for example deep ocean, deserts, mountains, and aquifers.
- Develop Red List-compatible assessment criteria for microbial communities, focusing on community integrity, functional collapse, and habitat specificity as well as vulnerability, and in certain circumstances on the extinction threat for keystone or specialist species.
- Construct Community Integrity Indices to monitor the health and resilience of microbial ecosystems using metrics such as taxonomic and functional diversity, functional redundancy, and sensitivity to disturbance.

2) Planning. Robust microbial conservation requires actionable guidance rooted in both ecological science and practical implementation.

- Create microbial conservation planning templates including costing tools for *in situ* and *ex situ* interventions, from habitat restoration and rewilding to microbiome and species banking.
- Co-develop risk–benefit economic frameworks for microbial interventions, including the use of probiotics, engineered microbes, and transplants in conservation programs. These frameworks should include holistic assessments of sustainability, feasibility, and potential unintended consequences, ensuring effective deployment that is aligned with safety, efficacy, and local context. It will be important to include environmental impact assessments, adaptive management frameworks, risk–benefit analyses, and life cycle assessments. Similar activities accounting for microbiome modifications could ensure actions are safe, effective, and context-appropriate. This will facilitate the development of microbial management recommendations for inclusion in protected area planning, especially where microbial ecosystems underpin host species viability and ecological resilience.
- Co-develop ethical frameworks to coordinate the needs and priorities of multiple communities and stakeholders in conservation efforts, which can serve as templates for creating these collaborative networks, gathering their feedback, stimulating discussion and cooperation within the network, and creating an action plan which adheres to the best practices, similar to the ethos of codes of conduct for scientific and medical research.

3) Action. The MCSG will serve as a catalyst for on-the- ground conservation and restoration efforts where microbial ecosystems are central to success.

- Develop pilot programs, coordinated with relevant existing IUCN efforts, that use microbial solutions to restore degraded ecosystems and threatened species, such as managing microbiomes to protect and restore coral reefs, reduce methane emissions while maintaining livestock productivity, deploying microbes to improve plant resilience to drought and heat, bioremediating polluted soils and waters, and stabilizing carbon and other soil health metrics in degraded lands.
- Analogous to successful global seed vaults (e.g. the Svalbard Global Seed Vault) and wildlife genetic repositories (such as the San Diego Zoo Wildlife Alliance’s Frozen Zoo®), microbial conservation efforts must align and accelerate activities that safeguard microbial diversity for future resilience and restoration. This includes the protection of natural microbial habitats, the systematic archiving of environmental and host-associated microbiome samples (especially those associated with threatened macroorganisms), and the expansion and integration of microbial biobanks and culture collections. These include leading facilities such as the Leibniz Institute DSMZ-German Collection of Microorganisms and Cell Cultures, American Type Culture Collection, the Japan Collection of Microorganisms, the China General Microbiological Culture Collection Center, and the Belgian Co-ordinated Collections of Microorganisms. Complementing these, newer initiatives such as the Microbiota Vault [37] aim to store uncultured and cryopreserved microbiota from diverse populations and environments, focusing on preserving microbial functions and community structures. By coordinating across these repositories and linking them with global conservation goals, the microbial conservation community can build a robust infrastructure for the long- term stewardship of microbial life.
- Build robust funding pipelines through philanthropy, public–private partnerships, and multilateral mechanisms, including the Global Environment Facility, the United Nations Soil Health Initiative, and bilateral conservation programs, to catalyze large-scale investment in microbial conservation and restoration as critical pillars of planetary, macrobial species, and human health. Promote equity and benefit-sharing in accordance with the UN CBD and its Nagoya Protocol in recognition of countries’ sovereign rights over their biodiversity as well as other UN instruments such as the Biodiversity Beyond National Jurisdiction and World Health Organization’s Pandemic Agreement, to ensure that microbial resources everywhere are accessed and used responsibly. This includes working with provider countries and indigenous communities and traditional knowledge holders to understand re- search priorities, guide methodological approaches, and shape the expected benefits of the work being returned to providers. This includes obtaining free, prior and informed consent under mutually agreed terms in an effort to increase the diversity of sources and environments for microbial biobanking (human populations, traditional foods, under-represented environments, etc.) Work with Indigenous peoples and local communities includes respecting the right of peoples *not* to share microbial communities.

4) Networking. Microbial conservation must be a globally inclusive movement, rooted in interdisciplinary collaboration.

- Engage a diverse, global membership, with active recruitment across all IUCN Commissions and regions and strong representation from low- and middle- income countries, where many unique microbial ecosystems and indigenous and traditional stewardship systems reside.
- Establish partnerships with professional societies (e.g. Applied Microbiology International, International Society for Microbial Ecology, American Society for Microbiology, and the International Union of Microbiological Societies), microbial biobank networks, and indigenous knowledge holders to co- produce knowledge and guide community-led conservation.
- Facilitate collaborations with other Conservation Specialist Groups of the IUCN, especially where microbes intersect with existing priorities—such as wildlife health, soil biodiversity, freshwater systems, and invasive species.
- Provide expertise to policy frameworks addressing microbial biodiversity value, such as the new Biodiversity Beyond National Jurisdiction agreement which will likely enter into force in 2026 and the further implementation of the Kunming-Montreal Global Biodiversity Framework (KMGBF).

5) Communication and Policy. Central to the MCSG’s mission is the imperative to change the narrative and actions around microbial life, from invisible, unvalued and irrelevant to indispensable.

- Launch the “Invisible but Indispensable” and “Tiny but Mighty” campaign, targeting conservation audiences, policymakers, and the general public to build awareness of microbial conservation imperatives.
- Develop policy briefs and white papers aligned with major global frameworks, including the CBD, KMGBF, Intergovernmental Science-Policy Platform on Biodiversity and Ecosystem Services, and One Health initiatives.
- Ensure microbial representation at international forums, including the CBD, UNFCCC COP, IUCN World Conservation Congress, High Seas Biodiversity Beyond National Jurisdiction negotiations, and regional biodiversity summits.
- Facilitate the expansion of microbial discovery programs in under sampled regions, as well as on developing standards for monitoring microbial biodiversity in the environmental context (e.g. metage- nomic/eDNA surveys)

Together, these objectives provide a roadmap for incorporating microbial life into biodiversity conservation. By protecting microbial communities, we secure the foundations of terrestrial and aquatic ecosystems, enable successful restoration, and safeguard the symbiotic microbiota that sustain plant, animal, and human life. The MCSG calls on the global conservation community to recognize that the microbial world is not a footnote; it is the foundation.

Integration with existing structures of the IUCN species survival commission.

The MCSG will adopt a hub-and-spoke model, maintaining liaison officers within other SSC groups (e.g. Climate Change, Wildlife Health, Coral Specialist Group). This will allow for the integration of microbial dimensions into broader conservation planning, ecosystem health assessments, and restoration monitoring. Moreover, the MCSG will work with Red List Authorities and the IUCN Red List of Ecosystems to adapt assessment criteria appropriate for microbial communities and functional ecosystems. We will also partner with foundations and NGOs already addressing threats to microbial biodiversity. In parallel, we will engage with IUCN’s World Commission on Environmental Law to explore the legal dimensions of microbial conservation, linking with existing initiatives such as the Rights of Nature and emerging efforts to recognize the Rights of Microbes (https://repository.graduateinstitute.ch/record/320234?v=pdf).

## Conclusion and call for participation

The creation of the IUCN MCSG represents a necessary and overdue step in global conservation. Without microbes, no conservation goal, from reforestation to species survival, can be sustainably achieved. Recognizing microbial diversity as an integral part of biodiversity is not only scientifically valid but practically essential for securing the ecological, health, and economic futures of our planet. We welcome microbiologists, ecologists, conservationists, health professionals, citizen scientists, indigenous communities, and policy stakeholders to support and collaborate with us in this transformative mission.
